# Kebab: Kinetochore and EB1 Associated Basic Protein That Dynamically Changes Its Localisation during *Drosophila* Mitosis

**DOI:** 10.1371/journal.pone.0024174

**Published:** 2011-09-02

**Authors:** Ana M. Meireles, Nikola S. Dzhindzhev, Hiroyuki Ohkura

**Affiliations:** The Wellcome Trust Centre for Cell Biology, School of Biological Sciences, The University of Edinburgh, Edinburgh, United Kingdom; Duke University Medical Center, United States of America

## Abstract

Microtubule plus ends are dynamic ends that interact with other cellular structures. Microtubule plus end tracking proteins are considered to play important roles in the regulation of microtubule plus ends. Recent studies revealed that EB1 is the central regulator for microtubule plus end tracking proteins by recruiting them to microtubule plus ends through direct interaction. Here we report the identification of a novel *Drosophila* protein, which we call Kebab (kinetochore and EB1 associated basic protein), through *in vitro* expression screening for EB1-interacting proteins. Kebab fused to GFP shows a novel pattern of dynamic localisation in mitosis. It localises to kinetochores weakly in metaphase and accumulates progressively during anaphase. In telophase, it associates with microtubules in central-spindle and centrosomal regions. The localisation to kinetochores depends on microtubules. The protein has a domain most similar to the atypical CH domain of Ndc80, and a coiled-coil domain. The interaction with EB1 is mediated by two SxIP motifs but is not required for the localisation. Depletion of Kebab in cultured cells by RNA interference did not show obvious defects in mitotic progression or microtubule organisation. Generation of mutants lacking the *kebab* gene indicated that Kebab is dispensable for viability and fertility.

## Introduction

The microtubule cytoskeleton is a dynamic network, constantly reorganising itself in response to various internal and external cues. In order to perform cellular functions as diverse as chromosome segregation, flagellar movement or neuronal transport, the microtubule network needs complex regulatory mechanisms [1]. Even though microtubule-associated proteins (MAPs) are regarded as the main regulators of microtubule organisation and dynamics [1], our knowledge of MAPs is still limited. Unlike microtubule motors, most non-motor MAPs do not have easily recognisable features within their primary sequence or high sequence conservation across eukaryotes. Furthermore, as hundreds of MAPs interact with microtubules even in a single cell, functional redundancies are likely to be very high. As the behaviour of microtubules varies within cells, and in different cell cycle stages and cell types, MAPs must be spatially and temporally regulated. Therefore we are still a long way from knowing the full complement of MAPs, how they regulate microtubules, and how they themselves are regulated in cells.

The microtubule is a polar filament made of tubulin dimers. The two ends, called plus and minus ends, behave differently from each other *in vitro* and *in vivo* [2]. The plus end is much more dynamic, and often interacts with other cellular structures, such as kinetochores and the cell cortex. Critically the interaction with other cellular structures influences the behaviour of the microtubule plus ends [3]. Therefore, microtubules can read the cellular environment to adopt an organisation specific to cell function. A specialised group of MAPs has been found to bind preferentially to microtubule plus ends, and are collectively called plus-end tracking proteins [3]. Plus-end tracking proteins are considered to be important for regulation of microtubule plus ends, and therefore have drawn much attention in recent years.

Recent studies highlighted the central role of EB1 among plus-end tracking proteins [4]. EB1 was originally identified as a binding partner of a tumour suppressor protein, adenomatous polyposis coli (APC), and later shown to track microtubule plus ends in cells [5], [6]. EB1 is a highly conserved protein across yeasts to humans [7], and is required for proper regulation of microtubule plus ends [4]. The central role for EB1 in microtubule plus end regulation has been demonstrated, as EB1 can track microtubule plus ends in the absence of other proteins *in vitro*, it physically interacts with many microtubule plus end tracking proteins, and is required for recruitment of these proteins to the microtubule plus ends *in vivo* [8], [9], [10], [11].

The conserved C-terminal domain of EB1 interacts with other microtubule plus end tracking proteins [12]. So far two motifs, the CAP-Gly domain and the linear motif SxIP, have been identified to interact with EB1 [13], [14]. However, interactions between EB1 and other microtubule plus end tracking proteins are very dynamic, and are usually undetectable by co-immunoprecipitation from cell extracts [15]. Nevertheless, proteins interacting with EB1 have been successfully identified by mass-spectrometry after pull-down from cell extract using bacterially produced EB1 proteins [16], [17], [18], [19], [20]. These EB1 interacting proteins have been shown to play important roles in various aspects of microtubule regulation [17]. Further identification of EB1 interacting proteins will be crucial for a full understanding of microtubule regulation.

Here we report the identification of a novel EB1 interacting protein, which we call Kebab, from *Drosophila melanogaster*, by an *in vitro* expression screen. We found that Kebab shows a unique dynamic localisation during mitotic progression. It localises to kinetochores during mitosis, where it progressively accumulates during anaphase. In telophase it associates with microtubules. Kebab interacts with EB1 through two SxIP motifs, but this interaction is not required for Kebab localisation. Kebab is dispensable for viability and fertility in flies.

## Results

### Identification of a novel EB1-interacting protein, Kebab, in *Drosophila*

To gain further insight into the regulation of microtubule plus ends, we have identified a novel EB1-interacting protein, CG31672, that associates with mitotic kinetochores. The gene is located at 22C1 on chromosome arm 2L, and the predicted molecular weight of the protein is 64 kDa. As it is a very basic (pI = 9.7), we called this previously uncharacterised protein Kebab (abbreviated as to Keb), which stands for kinetochore and EB1 associated basic protein.

**Figure 1:**
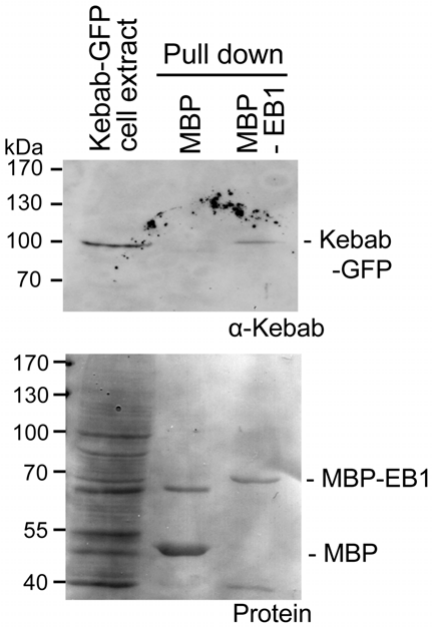
Kebab interacts with EB1. Cell extract from a stable cell line expressing Kebab-GFP was incubated with bacterially produced MBP and MBP-EB1. MBP and MBP-EB1 were pulled down and subjected to western blot using an anti-Kebab antibody (the upper panel). One twentieth of the cell extract was run, relative to the pull-down fractions. Protein staining of the same membrane is shown in the lower panel. Kebab-GFP is specifically pulled down with EB1.

We identified this EB1-interacting protein through *in vitro* expression cloning using a collection of unique annotated *Drosophila* cDNAs (DIVEC; [21]). In brief, cDNAs from the collection were transcribed and translated *in vitro*. Bacterially produced MBP (maltose binding protein) alone or MBP-EB1 was incubated with the translated product, and pulled down to assay specific interaction with EB1. These putative EB1-interacting proteins were further examined for their subcellular localisation in a *Drosophila* cultured S2 cell line originated from embryos. Because of the unique localisation, Kebab was selected for further study. Interaction between Kebab protein and EB1 was further confirmed by pull down of Kebab from S2 cell extract by MBP-EB1 (Figure 1).

### Kebab associates with kinetochores and spindle microtubules

**Figure 2:**
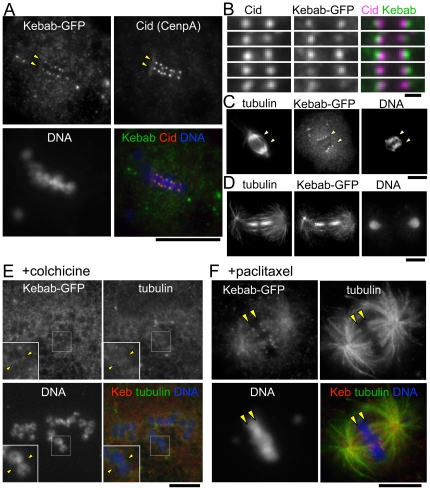
Kebab localises to kinetochores and spindle microtubules. (A) Kebab localises to mitotic kinetochores. S2 cells were transfected with a plasmid expressing Kebab-GFP under the actin promotor, and immunostained for GFP, Cid and DNA. The arrowheads indicate the position of centromeres. Bar = 10 µm. (B) Kebab foci were located outside of CID foci. Magnified images of sister centromeres. Bar = 1 µm. (C) Kebab localises to kinetochores in anaphase. S2 cells expressing Kebab-GFP were immunostained for α-tubulin, GFP and DNA. Bar = 10 µm. (D) Kebab localises to spindle microtubules in telophase. Bar = 10 µm. (E) S2 cells expressing Kebab-GFP were incubated with colchicine and immunostained. In the inserts, the boxed region containing a single chromosome was magnified. The arrowheads indicate the position of kinetochores at the primary constriction. Kinetochore signals were greatly reduced. Bar = 10 µm. (F) S2 cells expressing Kebab-GFP were incubated with paclitaxel and immunostained. The arrowheads indicate the position of kinetochores. Bar = 10 µm.

To analyse its subcellular localisation, Kebab was fused to GFP and expressed in a *Drosophila* cultured S2 cell line. Kebab protein fused to GFP showed dynamic localisation to the mitotic apparatus during mitosis. The localisation is identical using both N and C-terminal GFP fusions, suggesting it reflects the native localisation of Kebab protein. During mitosis, it is localised to multiple foci on chromosomes, which possibly correspond to kinetochores (Figure 2). To confirm this possibility, Kebab fused with GFP was co-stained with the centromere protein Cid (the *Drosophila* CenpA homologue). GFP foci were found to overlap with Cid foci (Figure 2A). Closer inspection showed that Kebab-GFP foci were located slightly outside each pair of Cid foci in metaphase, indicating Kebab is associated to kinetochores (Figure 2B). Kebab stayed on kinetochores during anaphase, and the signals appeared to intensify in late anaphase (Figure 2C). From late anaphase to telophase, it was also associated with residual spindle microtubules between the separated chromosomes (Figure 2D). In interphase, it localised to the cytoplasm and was concentrated around the nucleus (Figure S1).

To test whether Kebab localisation to kinetochores depends on microtubules, cells were incubated with a high dose of colcemid or colchicine to depolymerise all microtubules in mitotic cells. In both cases, after immunostaining we found that the signals on kinetochores were greatly reduced to an undetectable level (Figure 2E). To test whether the stabilisation of microtubules influences the kinetochore localisation, cells were incubated with paclitaxel. In this case, we found that the signals on kinetochores and microtubules were increased (Figure 2F). These results demonstrate that microtubules are required for kinetochore localisation of Kebab.

### Dynamic localisation of Kebab during mitosis

**Figure 3:**
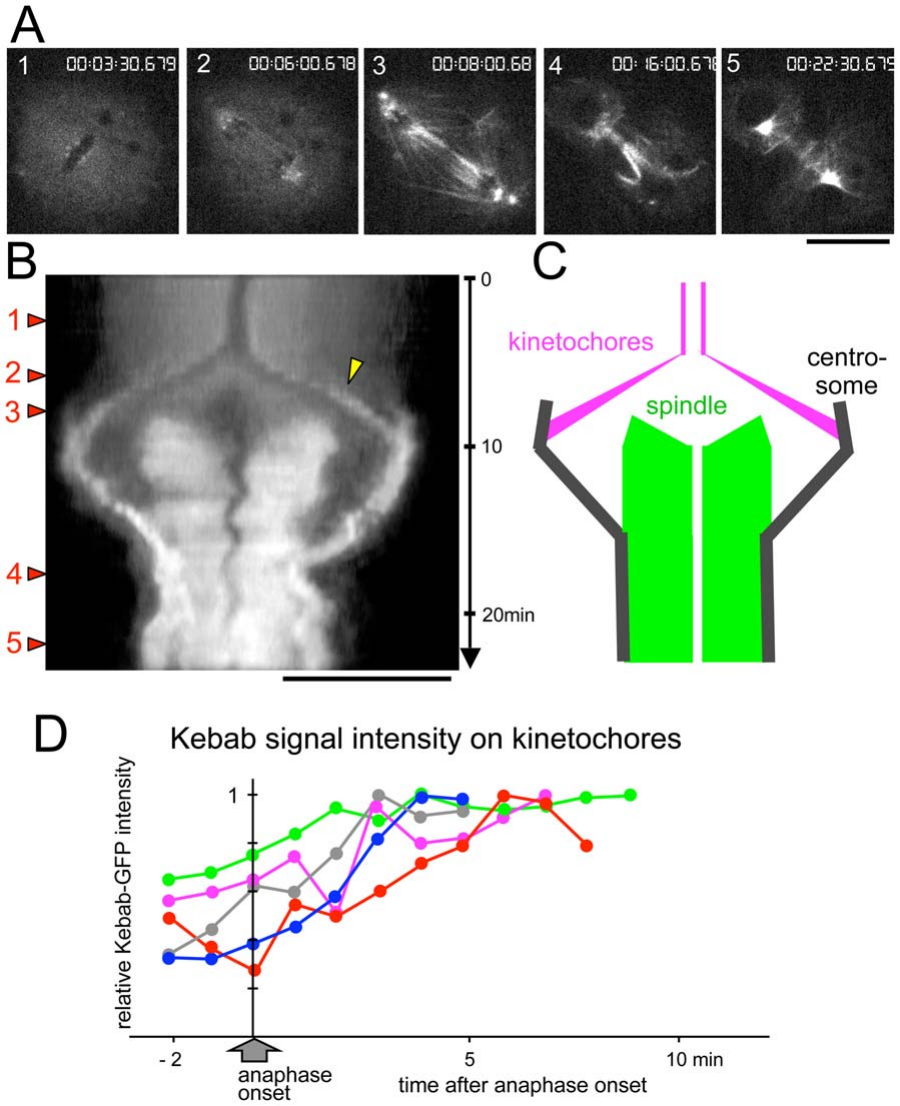
Kebab localisation dynamically change during mitosis. S2 cells stably expressing Kebab-GFP and mCherry-α-tubulin were observed under a spinning disc confocal microscope. Bars = 10 µm. (A) Still images of Kebab-GFP localisation at different stages of mitosis are indicated on the left side of B. (B) A kymograph of maximum intensity projection with the long axis of the spindle (X-axis) against time (Y-axis). Arrowhead indicates accumulating kinetochore signals during anaphase. (C) A diagram of the kymograph in B indicating cellular location of signals. (D) Increase of Kebab signal intensity on kinetochores during anaphase. Kebab signal intensities on kinetochores above the background were plotted from late metaphase to the end of anaphase for 5 random cells. The intensity values were normalised against the maximal value in each anaphase.

To follow the changing localisation of Kebab during mitosis in live cells, a stable cell line simultaneously expressing Kebab-GFP and mCherry-α-tubulin was generated and observed under a spinning disc confocal microscope (Movie S1; Figure 3A–C). In metaphase, Kebab localised to kinetochores, although the signal was weak (1 in Figure 3A). During anaphase, it progressively accumulated on kinetochores (2 in Figure 3A,B). In late anaphase, it also started to localise to microtubules notably in the central spindle and centrosomal regions (3 in Figure 3A,B). The intensity increased further when the cell entered telophase (4,5 in Figure 3A,B).

To further analyse the accumulation of Kebab on kinetochores during anaphase, the intensity of Kebab-GFP on kinetochores was quantified over time. Small equal-sized circles were drawn around kinetochores and in the cytoplasm, and the total pixel intensity within each circle was measured. The mean values of kinetochore signals above the cytoplasmic signal were plotted against time after normalisation. Although patterns of accumulation are not identical, the signals gradually increased throughout anaphase in all cells examined (Figure 3D).

### Kebab has an atypical CH domain, a coiled-coil region and EB1 binding motifs

**Figure 4:**
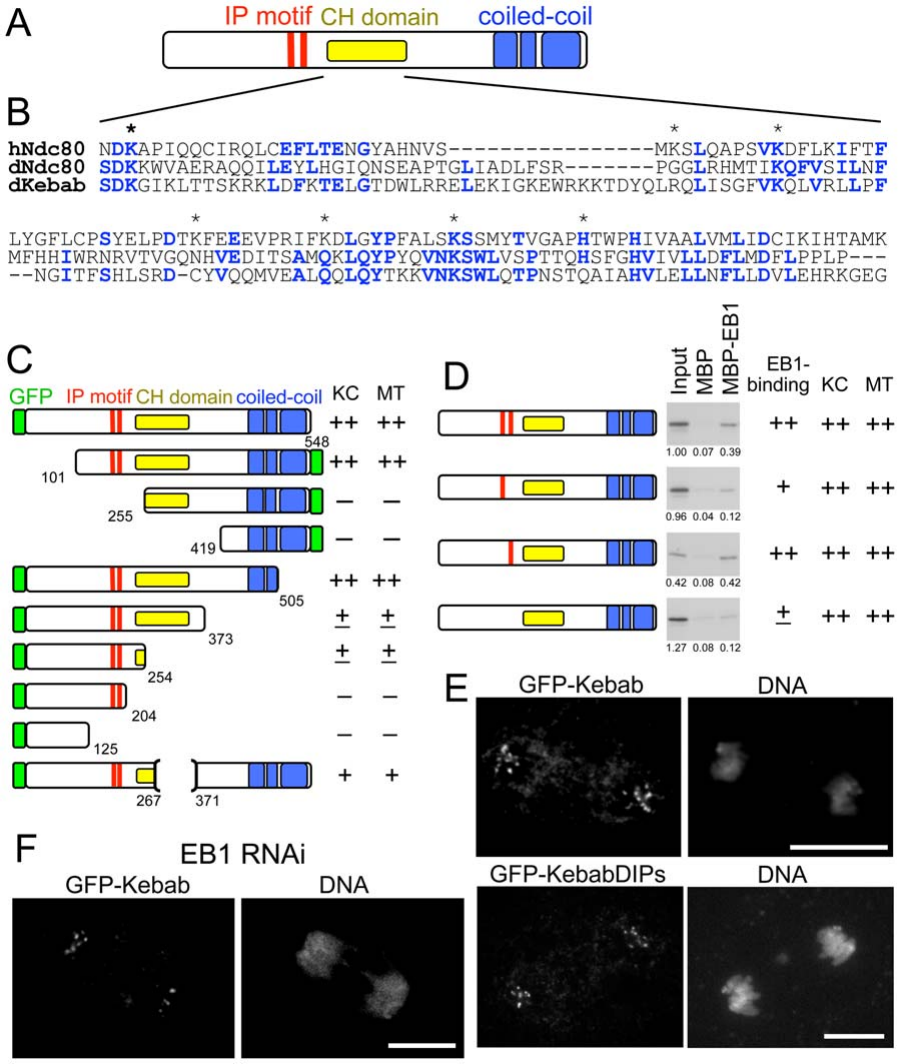
Kebab has EB1 binding motifs, an atypical CH domain and a coiled-coil region. (A) A diagram of the Kebab protein structure. (B) Similarity between the CH domains of human Ndc80 (hNdc80), *D. melanogaster* Ndc80 (dNdc80) and *D. melanogaster* Kebab (dKebab). The identical residues between two proteins were shown in bold blue letters. The asterisks indicate the residues important for the microtubule binding in human Ndc80. (C) A series of truncations and mutations tested for their localisation. KC and MT indicates the degree of localisation to kinetochores and microtubules. (++) full localisation, (+) weak localisation, (±) a trace of localisation, (−) no localisation. (D) EB1 binding assay of Kebab with mutated SxIP motifs. Radiolabelled proteins were *in vitro* translated and mixed with beads coupled with MBP and MBP-EB1. Pull-down fractions were run along with the original input (25% of pull-down fractions) and radiolabelled Kebab was detected by autoradiograph. Specific EB1 binding activity by this assay is indicated together with kinetochore or microtubule localisation (KC or MT). (E) Kinetochore localisation of a full-length GFP-Kebab and Kebab with both EB1 binding motifs mutated (ΔIPs). (F) Kinetochore localisation in EB1 depleted cells. Bar = 10 µm.

To gain further insight into the Kebab protein, we investigated the existence of structural domains in Kebab using bioinformatic tools (Figure 4A). Clear orthologues which share a significant homology to Kebab over the entire region were found in the genomes of *Drosophila* species, but not beyond. The middle region (amino acids 238–373) was found to have a weak but significant similarity to the calponin homology (CH) domain of the human kinetochore protein Ndc80 and its orthologues (Figure 4B), but not to CH domains of other proteins. The CH domains of Ndc80 are well diverged from other CH domains at the primary sequence level. The homology was only recognised after determination of crystal structures, and is considered to contribute to microtubule binding [22], [23]. The residues of the human Ndc80 CH domain involved in microtubule binding (asterisks in Figure 3B; [23]) are often conserved in Kebab. Therefore this domain of Kebab and the CH domain of Ndc80 appear to form a subfamily of CH domains.

In addition to this CH domain, extensive coiled coils were predicted in the C-terminal residues (385–411aa; 436–456aa; 513–539aa). Kebab has two regions near the CH domain (at 150, 169) which match an SxIP motif (S/TxIP) which is a known EB1 binding motif (Figure 4A). The other known EB1 binding motif, CAP-Gly domain [13], was not found.

### Kinetochore and microtubule localisation is independent of EB1 binding

To define the regions responsible for the localisation of Kebab, a series of truncated proteins with a GFP tag were expressed in S2 cells and examined for their localisation by immunostaining using an anti-GFP antibody (Figure 4C, D; Figure S2). A series of truncations at the N-terminus suggested that the region between the 101st and 254th residues is important for both kinetochore and microtubule localisation. A series of truncations at the C-terminus revealed that the N-terminal 254 residues are sufficient for minimal localisation to kinetochores and microtubules. Kebab lacking most (268–370) of the CH domain was still able to bind to kinetochores and microtubules, indicating that a full CH domain is not essential for localisation.

It is possible that the two potential EB1 binding motifs (SxIP) located close to the CH domain may be involved in microtubule or kinetochore localisation. To experimentally test whether these SxIP motifs are responsible for EB1 binding of Kebab, a mutation was introduced to one or both of the SxIP motifs in a full-length protein. The mutants, together with the wild-type protein, were translated *in vitro* and tested for binding to MBP-EB1 and MBP alone by pull down assay (Figure 4D). A mutation in the first motif greatly reduced the binding to EB1, while a mutation in the second one slightly reduced it. Double mutants further reduced EB1 binding to the minimum level. These results showed that the two SxIP motifs function additively in the interaction with EB1 and the motifs together contribute to most, if not all, of Kebab's interaction with EB1.

Next, to test the contribution of EB1 interaction for the localisation, a full-length Kebab with a mutation in SxIP motifs was expressed in S2 cells (Figure 4C,E). Kinetochore localisation of Kebab was unaffected by either single mutations or the double mutation. Furthermore, EB1 depletion by RNAi did not change kinetochore localisation of Kebab (Figure 4F). This showed that Kebab kinetochore localisation does not require EB1 interaction.

### Kebab is dispensable for mitotic progression

**Figure 5:**
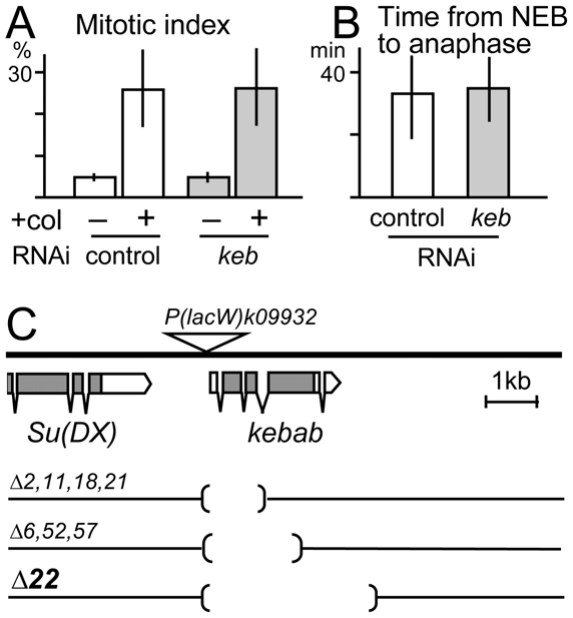
Kebab is dispensable for mitotic progression and fly viability and fertility. (A) Mitotic index of S2 cells without or after colchicine incubation. S2 cells were subjected to RNAi of *kebab* and a control. S2 cells were immunostained for phospho-H3 at Serine 10 and DNA. The mitotic index was the proportion of phospho-H3 positive cells. Bars indicate standard deviations. (B) Time from nuclear envelope breakdown to anaphase onset. S2 cells expressing mCherry-RCC1 were subjected to RNAi of *kebab* and a control, and observed in live cells. (C) The genomic region around *kebab*. The open boxes, grey boxes and kinked lines indicate non-coding exons, coding exons and introns, respectively. The triangle indicates the position of the P-element insertion in *k09932*. The parentheses indicate regions deleted in each *keb* mutant.

To understand its cellular role, Kebab was depleted from S2 cells by RNA interference (RNAi). RNAi using double-stranded RNA (dsRNA) is known to work robustly in S2 cells [24], [25]. The effectiveness of RNAi was confirmed by western blots on a cell line expressing Kebab-GFP treated with the dsRNAs (Figure S3). Three dsRNAs, each corresponding to non-overlapping regions of the *kebab* transcript were used and gave essentially identical results. These cells were immunostained for α-tubulin, mitotic specific phospho-H3 (at serine 10) and DNA. The mitotic index was calculated as the frequency of phospho-H3 positive cells. Each mitotic figure was categorised and counted. These studies did not reveal a significant difference between *kebab* and control RNAi (Figure 5A).

To test the involvement of Kebab in the spindle checkpoint, cells depleted of Kebab were challenged by incubation with colchicine, a microtubule depolymerising drug. In control cells, the mitotic index increased after incubation with colchicine. In Kebab depleted cells, the mitotic index also increased to a level similar to the control after colchicine incubation (Figure 5A).

To detect more subtle defects in the timing of mitotic progression, live imaging was carried out using cells stably expressing GFP-α-tubulin. The cells were treated with dsRNA for *kebab* or a control. The time was measured from nuclear envelope breakdown to the onset of anaphase (Figure 5B). There was no significant difference between *kebab* RNAi and the control.

Although no firm conclusions can be drawn from RNAi experiments, these studies suggest that Kebab is dispensable for mitotic progression in S2 cells.

### Flies lacking Kebab are viable and fertile

To understand the role of Kebab in developing flies, deletion mutants of Kebab were generated. We took advantage of a line in which a P-element is inserted into the 5′ non-coding region of the *kebab* gene. The P-element was remobilised and the presence/absence of the genomic regions surrounding the P-element was examined by PCR. We have isolated a mutant (Δ*22*) in which the *kebab* coding region is completely deleted without affecting neighbouring genes (Figure 5C; Figure S4). In addition, multiple lines lacking most of the *kebab* coding region were identified. These mutants, including one completely lacking the *kebab* gene, were viable and fertile in both sexes. Furthermore, a homozygous stock can be maintained without difficulty for many generations. These results demonstrated that Kebab is not essential for viability and fertility.

To genetically test whether chromosomes are segregated accurately in the mutant, we examined segregation of the sex chromosomes in germ lines. As the sex in *Drosophila* is determined by the number of X chromosomes independently of the Y chromosome, aneuploidy in gametes would give rise to viable male progeny without the Y chromosome, or females with the Y chromosome. A *B^s^*-marked Y chromosome was used to monitor the inheritance of the Y chromosome.

When *keb* mutant females were crossed with wild type, aneuploidy in the viable progeny appeared more frequent (1.19±2.20% in *keb* vs 0.17±0.53% in wild type), but the difference is not statistically significant (p = 0.07). Also, when the *keb* deletion mutant males were crossed with wild type, the frequency of aneuploidy in the viable progeny was not significantly different from a control (0.18±0.68% in *keb* vs 0.89±1.22% in wild type; p = 0.07).

In conclusion, the removal of Kebab has little detrimental effect on a cultured cell line or in developing flies.

## Discussion

EB1 regulates microtubule plus ends through interaction with multiple proteins [26]. In this study we identified a novel EB1-interating protein, Kebab, which shows a dynamic localisation to kinetochores and microtubules in mitosis.

Previously several studies have successfully identified EB1-interacting proteins by mass-spectrometry after pull down using bacterially produced EB1 protein [20]. One drawback of this approach is that the chance of a protein being identified depends on its level in a particular tissue or cell line. We have identified Kebab by *in vitro* expression cloning. *In vitro* expression cloning was originally developed by Lustig and co-workers, using random cDNAs from *Xenopus* eggs [27], [28]. It was later adapted for use with a collection of annotated unique *Drosophila* cDNAs [21]. This study looked for substrates of a kinase by examining the shift in gel mobility after kinase reaction. We further adapted this method for a pull down assay to identify interacting proteins. The advantage of this approach over a mass-spectrometry based one would be that low abundance proteins or those expressed in specific cell types have equal chance of being identified, as long as cDNA has been isolated from some tissues.

We found that Kebab localises to kinetochores during mitosis. The relative position of Kebab to the *Drosophila* CenpA orthologue Cid, suggests it localises to outer kinetochores. The most notable feature of Kebab is that it progressively accumulates on kinetochores during anaphase. Although this behaviour is unusual among kinetochore proteins, a group of centromere proteins, including CenpA, CenpC and Mis18, have been reported to progressively accumulate on centromeres during anaphase [29], [30], [31]. These proteins interact with each other to define the centromere identity through loading of CenpA, a Histone 3 variant, on DNA [29]. It is still not understood why this occurs during anaphase. Interestingly, Kebab is not colocalised with CenpA, rather is localised outside of CenpA. Although all kinetochore proteins require CenpA for their localisation, generally they do not increase in intensity during anaphase. It is possible that Kebab recruitment to kinetochores may be somehow linked to CenpA loading, or the dose of CenpA on centromeres, rather than other kinetochore proteins. Alternatively, Kebab may be regulated by a previously unknown mechanism.

The second interesting property of Kebab is that microtubules are required for its kinetochore localisation. This property is unusual but not unique among kinetochore proteins. EB1 localisation to kinetochores in PtK1 cells was previously reported to depend on microtubules [32]. It was found to localise to only one of the sister kinetochores moving away from poles, which is coupled to microtubule polymerisation. In contrast, Kebab localisation appears to be symmetrical in metaphase and become more prominent when microtubules are depolymerising in anaphase. Other kinetochore proteins such as Ska1 and Ajuba are also reported to depend on microtubules for their localisation [33], [34]. The mechanism and significance of this microtubule dependency are still under speculation. Kebab may recognise kinetochore-associated microtubule plus ends regardless of the polymerisation state. Alternatively, Kebab is transported to kinetochores along microtubules. In either case, it is a very interesting and unusual property of a kinetochore protein.

The localisation of Kebab dynamically changes during mitotic progression. In late anaphase, Kebab starts localising to spindle microtubules or centrosomal regions. The association with microtubules becomes more prominent in telophase. This changing pattern of localisation is unique among previously reported kinetochore proteins or microtubule-associated proteins. Nevertheless, other types of proteins are known to change localisation during mitotic progression. For example, the chromosomal passenger complex localises to centromeres/kinetochores until metaphase and relocates to microtubules in the spindle midzone at the onset of anaphase [35]. This change in localisation is considered to be crucial for the change in kinetochore behaviour at the onset of anaphase, and stabilisation of the spindle midzone in telophase [35]. Dynamic localisation of Kebab may subtly influence a change in behaviour of kinetochore or spindle microtubules.

We showed that Kebab can directly bind to EB1 *in vitro*. Interaction with EB1 is mediated by two SxIP motifs located near the CH domain. The SxIP motif is a linear motif found in many EB1 binding proteins [14]. Mutations in both SxIP did not abolish the localisation of Kebab, suggesting interaction with EB1 is not essential for localisation. Consistently we found that EB1 depletion did not disrupt the localisation of Kebab. It is possible that EB1-independent localisation masks the EB1 dependent localisation in a specific location. Alternatively EB1 interaction may be important for Kebab function rather than the localisation. Further studies are needed to clarify the significance of EB1 interaction.

Kebab contains a domain which is most similar to the atypical CH domain of another kinetochore protein, Ndc80. Ndc80 is one of the critical proteins which connect kinetochores to microtubules [36]. This domain of Ndc80 is considered to be the microtubule binding domain [23]. The CH domain of Ndc80 is quite distinct from typical CH domains found in other proteins, and was only recognised after the crystal structure was determined [22]. Our discovery of the second member of this atypical CH domain group may shed light on how the essential kinetochore protein Ndc80 interacts with microtubules.

No obvious functions have been revealed by RNAi or generation of null mutants. Although there are no obvious paralogues in the *Drosophila melanogaster* genome, there may be other proteins that function redundantly with Kebab. Hundreds of proteins in each cell type can bind to microtubules, and collectively determine their behaviour. These consist of a diverse array of proteins, with only a small minority containing known microtubule binding motifs. It is likely that many structurally distinct proteins can function redundantly to regulate microtubules. For example, microtubule bundling can be achieved by many proteins or protein complexes which contain multiple microtubule binding sites. It is a challenge in biology to understand a system that involves many redundancies, such as microtubule regulation. Future identification of proteins that have overlapping function with Kebab will shed light on the function and regulation of Kebab protein in mitosis.

## Materials and Methods

### Molecular and protein techniques

Standard DNA manipulation and protein techniques were used [37]. The *kebab* coding region was introduced first into the Gateway entry vector pDONR221, and then into destination vectors, pAGW and pAWG to generate a plasmid for expression of Kebab fused to GFP either N or C-terminus under the actin5C promotor. Kebab deletions were created by PCR amplification of gateway expression clone with primers flanking the regions to be deleted and carrying an EcoRI site. Digestion with EcoRI and ligation generated the desired plasmid which was subsequently sequenced. Premature stop codons were introduced by site directed mutagenesis using Quick Change XLII site directed mutagenesis kit (Agilent), following manufacturer's instructions.

### Identification of Kebab

Kebab was identified by *Drosophila in vitro* expression cloning as described below. A pool of 12 cDNAs from a *Drosophila* Gold Gene Collection was transcribed and translated *in vitro* using the T7 TnT Quick Coupled system (Promega) in the presence of ^35^S-methionine (Easytag, Perkin Elmer). Each translated product was split into two and incubated in DIVEC buffer (50 mM Hepes pH 7.6, 1 mM MgCL_2_, 1 mM EGTA, 200 mM NaCl, 0.5% TritonX100) for 60 minutes with amylose resin (New England Biolabs) coupled with bacterially-produced MBP or MBP-EB1. After extensive washing in DIVEC buffer, the beads were boiled with the sample buffer and run on an SDS gel. Dried gels were exposed to X-ray film (Hyperfilm, GE Healthcare). cDNA pools which gave bands specific for MBP-EB1 pull down were further studied by testing sub-pools until a single responsible cDNA was identified.

### Cell culture

*Drosophila* Schneider S2 cells were cultured and RNA interference (RNAi) was performed according to published methods [25]. Plasmids were transfected using Effectene transfection reagent (Qiagen) following manual's instructions. A cell line stably expressing GFP was a kind gift from Ron Vale [38]. Cell lines expressing GFP-Kebab and/or mCherry-α-tubulin were established by basticidin selection (25 µg/ml) after co-transfection with resistance vector (pCoBlast, Invitrogen). Double-stranded RNA (dsRNA) corresponding to regions amplified by primer pairs (forward/reverse) 5′-GTAGTATGGCTAAATCGC-3′/5′-CTCTTTGAAAGTTCTTGG-3′, 5′-ACAATTACCAAGAATCTC-3′/5′-CTAAGGGCTTCCCTGGGG-3′, and 5′-GCGAACATAAGCCACATC-3′/5′-CAATTATACTGATACAAC-3′of *kebab* were used. dsRNA corresponding to *E. coli* β-lactamase was used as a control.

### EB1 pull-down assay

MBP and MBP-EB1 were bacterially produced, purified and bound to amylose resin (New England Biolabs). About 1×10^7^ S2 cells expressing Kebab-GFP were resuspended in DIVEC buffer (50 mM Hepes pH 7.6, 1 mM MgCl_2_, 1 mM EGTA, 200 mM NaCl, 0.5% TritonX100) and sonicated for 1 min in pulses of 1 s ON 5 s OFF. The extract was incubated with the beads for 1 hr at 4°C with rotation. After extensive washing in DIVEC buffer, the beads were boiled with the sample buffer and run on an SDS gel.

### Cytological analysis

Immunostaining of S2 cells was carried out and examined as previously described [39]. Briefly, S2 cells were plated on ConcanavalinA (ConA) coated coverslips and after 2 hrs were fixed with 90% methanol, 3% formaldehyde, 5 mM NaHCO_3_ pH 9 at −80°C. Cells treated with colchicine (2 µM, Sigma) and colcemid (5 µM, Sigma) were fixed after 2 hrs treatment. Cells treated with Paclitaxel (1 µM, ICN) were fixed after 10 or 30 min treatments. Antibodies against mouse α-tubulin (DM1A, 1∶200, Sigma), rabbit α-GFP (1∶500, Molecular Probes), mouse α-GFP (3E6, 1∶500, Molecular Probes), mouse α-Cid (1∶100, AbCam) and rabbit α -histone-H3-phosphate (1∶500, Upstate) were used as primary antibodies. Images were captured using a Zeiss Axioplan 2 microscope equipped with a CCD camera (Hamamatsu) controlled by Openlab software (Perkin Elmer).

### Live imaging

S2 cells were plated in ConA coated MatTek glass-bottom dishes, in culture media, for 2 hrs. Samples were examined at room temperature by a microscope (Axiovert; Carl Zeiss) attached to a spinning-disc confocal head (Yokogawa) using Volocity (PerkinElmer). Images were acquired once every 30 s (analysis of mitotic progression) or once every 60 s (analysis of Kebab accumulation at kinetochores). For quantification of Kebab at kinetochores, 4 circles of 0.7 µm^3^ were drawn and used to manually track the brightest kinetochores and to establish background values. After background subtraction, mean intensities were normalised against the highest value for each sample, and plotted against time. Time zero was set to anaphase onset. A kymograph was generated by making maximum intensity projections first onto the X-Y plane and then onto the long axis of the spindle for each time point, and aligning this one dimension data against time as the second dimension.

### Fly techniques

Standard fly techniques were used [40]. *w^1118^* was used as wild type in this study. *keb* mutants were generated by remobilisation of a P-element (*k09932*) inserted near the coding region. The transposase gene *Δ2–3* was introduced into *P(lacW)k09932* by crossing. Chromosomes which have lost the *w^+^* gene on the P-element were selected and tested over a deficiency uncovering the *keb* gene. No chromosomes lethal over the deficiency were isolated. Viable chromosomes were tested over the deficiency for the presence of the *keb* genomic region by PCR. Once the stocks were established, the breakpoints were determined by further PCR.

Frequencies of sex chromosome aneuploidy were genetically determined using *B^s^-* marked Y chromosomes. In brief, 21–42 wild-type or mutant females were individually crossed with otherwise wild-type males carrying a *B^s^*-marked Y chromosome and the progeny were counted for aneuploidy (B females or non-B males). In reciprocal crosses, wild-type females were individually crossed with wild-type or mutant males carrying the *B^s^*-marked Y chromosome. Frequencies of aneuploidy from individual crosses were calculated, and the averages and standard deviations were determined for wild-type and the mutant. The Wilcoxon test was used to estimate the statistical significance of differences between the mutant and wild type.

## Supporting Information

Movie S1 **S2 cells expressing Kebab-GFP and mCherry-α-tubulin.** See Figure 3 for details.
(MOV)

Figure S1 **Kebab localises to the cytoplasm in interphase.** S2 cells were transfected with a plasmid expressing Kebab-GFP under the actin promotor, and immunostained for GFP, α-tubulin and DNA. Bar = 10 µm.
(TIF)

Figure S2 **Various mutations and truncations affect Kebab localisation.** A series of truncations and mutations were tested for Kebab localisation, as outlined for Figure 4. A representative image for each construct is shown to highlight the presence or absence of kinetochore localisation.
(TIF)

Figure S3 **RNAi of** ***kebab*** **is effective.** S2 cells stably expressing Kebab-GFP were treated with the dsRNAs used in this study. A western blot was carried out using an anti-GFP antibody (the upper panel) and the same membrane was stained for protein (the lower panel). Expression construct for Kebab-GFP does not contain the endogenous *kebab* 3′UTR and therefore was resistant to RNAi using dsRNA (#3) corresponding to the *kebab* 3′UTR. The other dsRNAs (#2, #4) correspond to the *kebab* coding region, and effectively depleted Kebab-GFP.
(TIF)

Figure S4 **The** ***kebab*** **gene is deleted from **Δ***22*** **.** (A) A diagram showing the genomic region around the *kebab* gene. Thick bars indicate the regions which PCR primer pairs would amplify. (B) PCR was carried out to define the genomic region absent in a putative deletion line (Δ*22*) generated by remobilisation of the P-element *k09932*. Genomic DNA was prepared from a male fly with Δ*22* over the deficiency *Df(2L)ED125* lacking the entire region surrounding the *kebab* gene, together with a wild-type control (w) and other putative deletions. PCR was carried out using each primer pair shown in A. The regions b and c are missing from Δ*22*, but a and d are intact.
(TIF)

## References

[1] Galjart N (2010). Plus-end-tracking proteins and their interactions at microtubule ends.. Curr Biol.

[2] Cassimeris L (1993). Regulation of microtubule dynamic instability.. Cell Motil Cytoskeleton.

[3] Schuyler SC, Pellman D (2001). Microtubule “plus-end-tracking proteins”: The end is just the beginning.. Cell.

[4] Akhmanova A, Steinmetz MO (2008). Tracking the ends: a dynamic protein network controls the fate of microtubule tips.. Nat Rev Mol Cell Biol.

[5] Su LK, Burrell M, Hill DE, Gyuris J, Brent R (1995). APC binds to the novel protein EB1.. Cancer Res.

[6] Mimori-Kiyosue Y, Shiina N, Tsukita S (2000). The dynamic behavior of the APC-binding protein EB1 on the distal ends of microtubules.. Curr Biol.

[7] Tirnauer JS, Bierer BE (2000). EB1 proteins regulate microtubule dynamics, cell polarity, and chromosome stability.. J Cell Biol.

[8] Dzhindzhev NS, Rogers SL, Vale RD, Ohkura H (2005). Distinct mechanisms govern the localisation of Drosophila CLIP-190 to unattached kinetochores and microtubule plus-ends.. J Cell Sci.

[9] Bieling P, Laan L, Schek H, Munteanu EL, Sandblad L (2007). Reconstitution of a microtubule plus-end tracking system in vitro.. Nature.

[10] Bieling P, Kandels-Lewis S, Telley IA, van Dijk J, Janke C (2008). CLIP-170 tracks growing microtubule ends by dynamically recognizing composite EB1/tubulin-binding sites.. J Cell Biol.

[11] Dixit R, Barnett B, Lazarus JE, Tokito M, Goldman YE (2009). Microtubule plus-end tracking by CLIP-170 requires EB1.. Proc Natl Acad Sci U S A.

[12] Slep KC (2010). Structural and mechanistic insights into microtubule end-binding proteins.. Curr Opin Cell Biol.

[13] Hayashi I, Wilde A, Mal TK, Ikura M (2005). Structural basis for the activation of microtubule assembly by the EB1 and p150Glued complex.. Mol Cell.

[14] Honnappa S, Gouveia SM, Weisbrich A, Damberger FF, Bhavesh NS (2009). An EB1-binding motif acts as a microtubule tip localization signal.. Cell.

[15] Komarova Y, Lansbergen G, Galjart N, Grosveld F, Borisy GG (2005). EB1 and EB3 control CLIP dissociation from the ends of growing microtubules.. Mol Biol Cell.

[16] Berrueta L, Tirnauer JS, Schuyler SC, Pellman D, Bierer BE (1999). The APC-associated protein EB1 associates with components of the dynactin complex and cytoplasmic dynein intermediate chain.. Curr Biol.

[17] Rogers SL, Wiedemann U, Häcker U, Turck C, Vale RD (2004). Drosophila RhoGEF2 associates with microtubule plus ends in an EB1-dependent manner.. Curr Biol.

[18] Gu C, Zhou W, Puthenveedu MA, Xu M, Jan YN (2006). The microtubule plus-end tracking protein EB1 is required for Kv1 voltage-gated K+ channel axonal targeting.. Neuron.

[19] Geraldo S, Khanzada UK, Parsons M, Chilton JK, Gordon-Weeks PR (2008). Targeting of the F-actin-binding protein drebrin by the microtubule plus-tip protein EB3 is required for neuritogenesis.. Nat Cell Biol.

[20] Grigoriev I, Gouveia SM, van der Vaart B, Demmers J, Smyth JT (2008). STIM1 is a MT-plus-end-tracking protein involved in remodeling of the ER.. Curr Biol.

[21] Lee LA, Lee E, Anderson MA, Vardy L, Tahinci E (2005). Drosophila genome-scale screen for PAN GU kinase substrates identifies Mat89Bb as a cell cycle regulator.. Dev Cell.

[22] Wei RR, Al-Bassam J, Harrison SC (2007). The Ndc80/HEC1 complex is a contact point for kinetochore-microtubule attachment.. Nat Struct Mol Biol.

[23] Ciferri C, Pasqualato S, Screpanti E, Varetti G, Santaguida S (2008). Implications for kinetochore-microtubule attachment from the structure of an engineered Ndc80 complex.. Cell.

[24] Clemens JC, Worby CA, Simonson-Leff N, Muda M, Maehama T (2000). Use of double-stranded RNA interference in Drosophila cell lines to dissect signal transduction pathways.. Proc Natl Acad Sci U S A.

[25] Rogers SL, Rogers GC, Sharp DJ, Vale RD (2002). Drosophila EB1 is important for proper assembly, dynamics, and positioning of the mitotic spindle.. J Cell Biol.

[26] Vaughan KT (2005). TIP maker and TIP marker; EB1 as a master controller of microtubule plus ends.. J Cell Biol.

[27] Lustig KD, Stukenberg PT, McGarry TJ, King RW, Cryns VL (1997). Small pool expression screening: identification of genes involved in cell cycle control, apoptosis, and early development.. Methods Enzymol.

[28] King RW, Lustig KD, Stukenberg PT, McGarry TJ, Kirschner MW (1997). Expression cloning in the test tube.. Science.

[29] Fujita Y, Hayashi T, Kiyomitsu T, Toyoda Y, Kokubu A (2007). Priming of centromere for CENP-A recruitment by human hMis18alpha, hMis18beta, and M18BP1.. Dev Cell.

[30] Jansen LE, Black BE, Foltz DR, Cleveland DW (2007). Propagation of centromeric chromatin requires exit from mitosis.. J Cell Biol.

[31] Lagana A, Dorn JF, De Rop V, Ladouceur AM, Maddox AS (2010). A small GTPase molecular switch regulates epigenetic centromere maintenance by stabilizing newly incorporated CENP-A.. Nat Cell Biol.

[32] Tirnauer JS, Canman JC, Salmon ED, Mitchison TJ (2002). EB1 targets to kinetochores with attached, polymerizing microtubules.. Mol Biol Cell.

[33] Hanisch A, Silljé HH, Nigg EA (2006). Timely anaphase onset requires a novel spindle and kinetochore complex comprising Ska1 and Ska2.. EMBO J.

[34] Ferrand A, Chevrier V, Chauvin JP, Birnbaum D (2009). Ajuba: a new microtubule-associated protein that interacts with BUBR1 and Aurora B at kinetochores in metaphase.. Biol Cell.

[35] Ruchaud S, Carmena M, Earnshaw WC (2007). Chromosomal passengers: conducting cell division.. Nat Rev Mol Cell Biol.

[36] Tanaka TU, Desai A (2008). Kinetochore-microtubule interactions: the means to the end.. Curr Opin Cell Biol.

[37] Sambrook J, Fritsch EF, Maniatis T (1989). Molecular Cloning: A Laboratory Manual.

[38] Goshima G, Vale RD (2003). The roles of microtubule-based motor proteins in mitosis: comprehensive RNAi analysis in the Drosophila S2 cell line.. J Cell Biol.

[39] Meireles AM, Fisher KH, Colombié N, Wakefield JG, Ohkura H (2009). Wac: a new Augmin subunit required for chromosome alignment but not for acentrosomal microtubule assembly in female meiosis.. J Cell Biol.

[40] Ashburner M, Golic KG, Hawley RS (2005). *Drosophila*: a laboratory handbook..

