# Supplementary figures and images for: Kebab: Kinetochore and EB1 Associated Basic Protein That Dynamically Changes Its Localisation during Drosophila Mitosis
Source: PLoS One. 2011 Sep 2;6(9):e24174. doi: 10.1371/journal.pone.0024174 (PMC3166307; doi:10.1371/journal.pone.0024174)

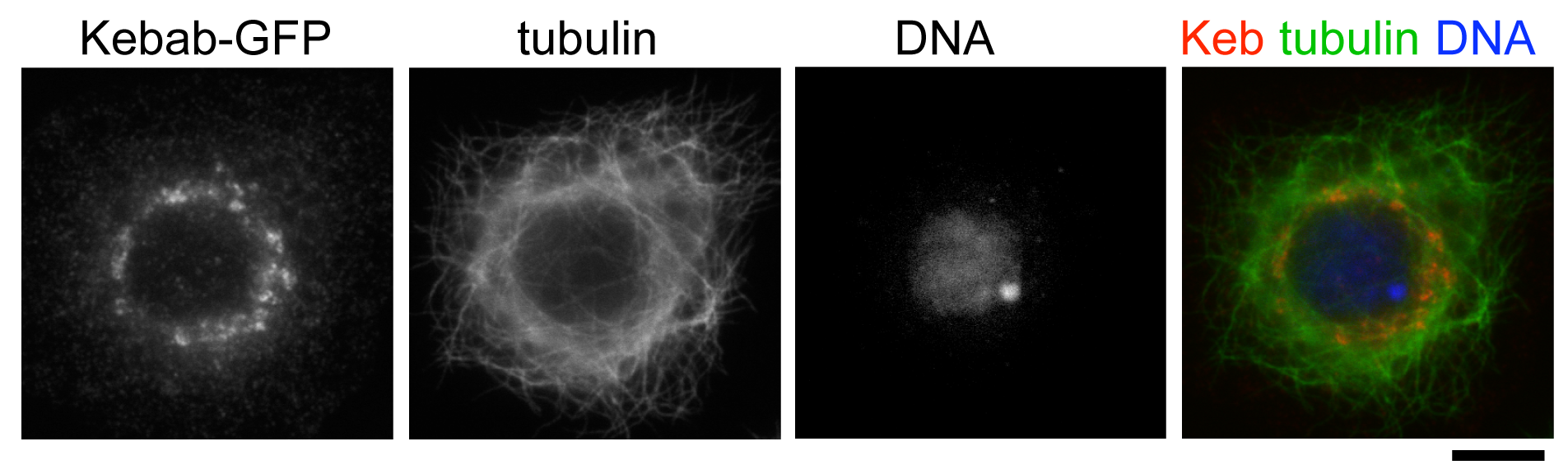

Supplement: Figure S1 — Kebab localises to the cytoplasm in interphase. S2 cells were transfected with a plasmid expressing Kebab-GFP under the actin promotor, and immunostained for GFP, α-tubulin and DNA. Bar = 10 µm. (TIF) [file pone.0024174.s002.tif]

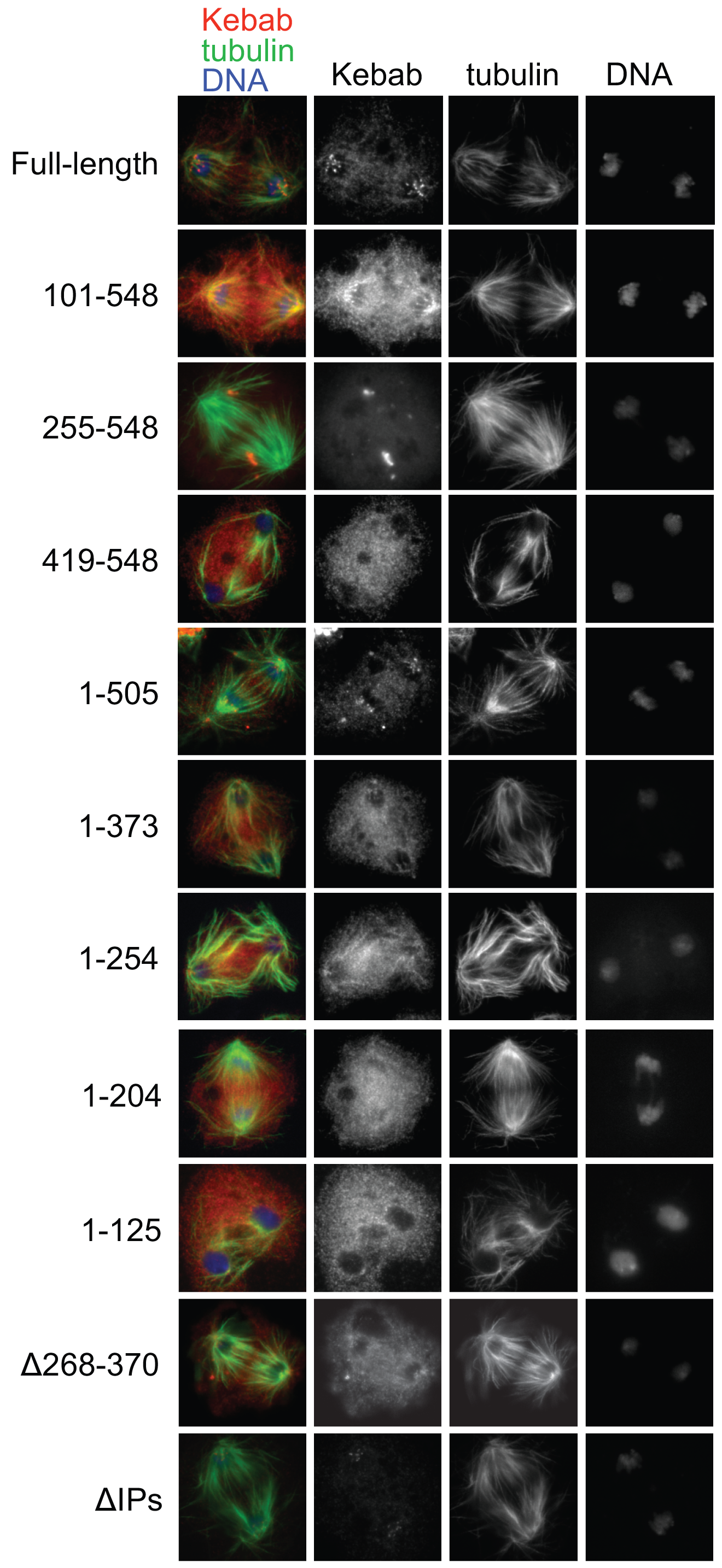

Supplement: Figure S2 — Various mutations and truncations affect Kebab localisation. A series of truncations and mutations were tested for Kebab localisation, as outlined for Figure 4. A representative image for each construct is shown to highlight the presence or absence of kinetochore localisation. (TIF) [file pone.0024174.s003.tif]

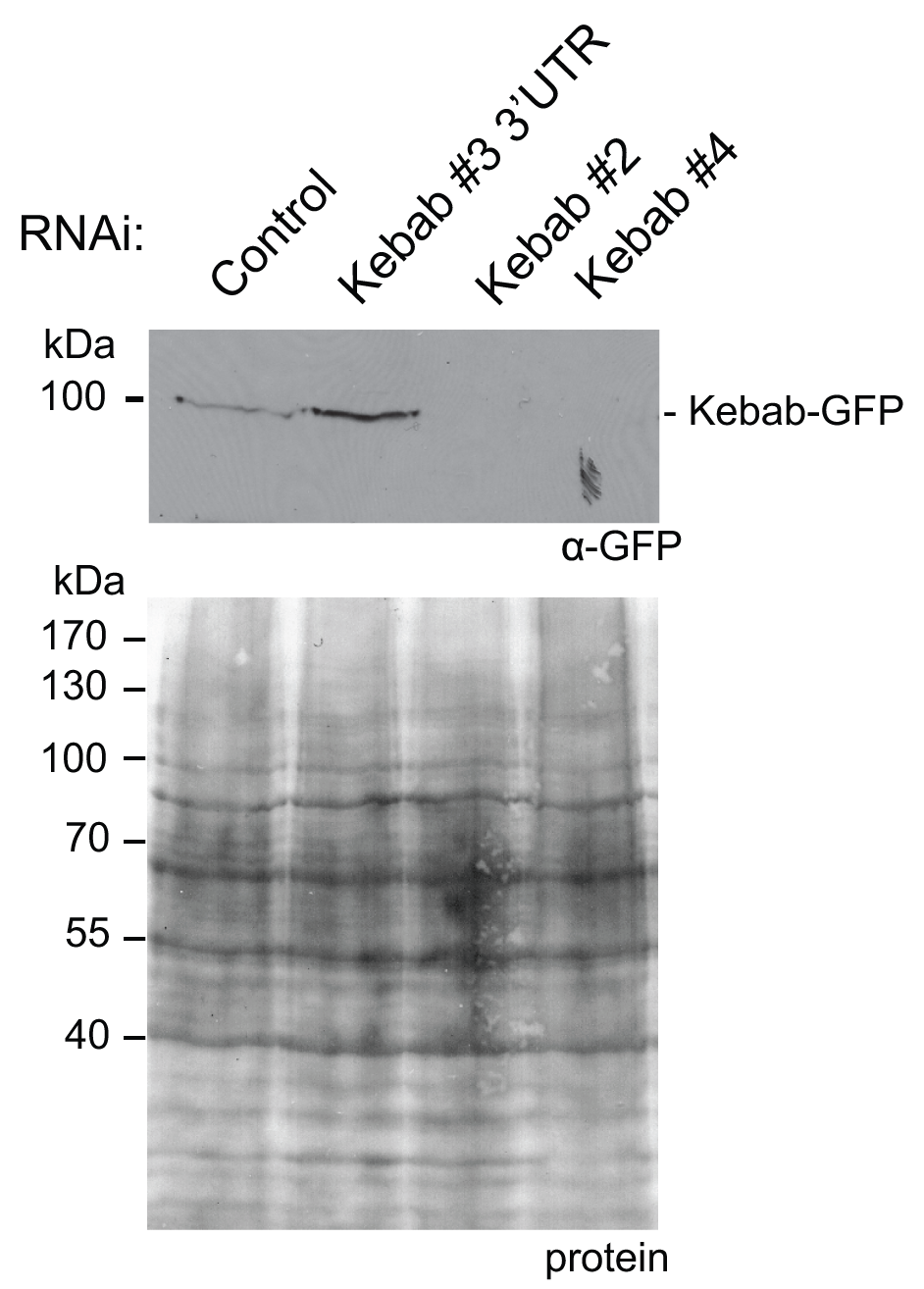

Supplement: Figure S3 — RNAi of kebab is effective. S2 cells stably expressing Kebab-GFP were treated with the dsRNAs used in this study. A western blot was carried out using an anti-GFP antibody (the upper panel) and the same membrane was stained for protein (the lower panel). Expression construct for Kebab-GFP does not contain the endogenous kebab 3′UTR and therefore was resistant to RNAi using dsRNA (#3) corresponding to the kebab 3′UTR. The other dsRNAs (#2, #4) correspond to the kebab coding region, and effectively depleted Kebab-GFP. (TIF) [file pone.0024174.s004.tif]

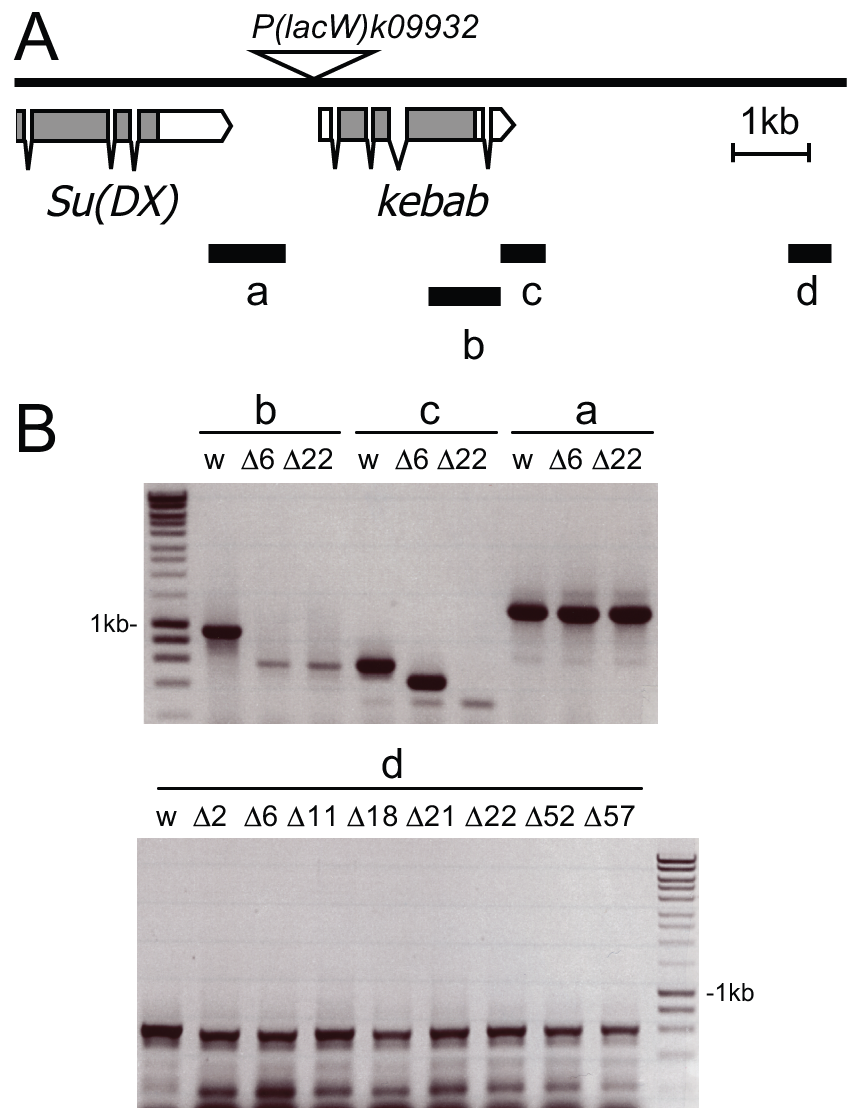

Supplement: Figure S4 — The kebab gene is deleted from Δ22 . (A) A diagram showing the genomic region around the kebab gene. Thick bars indicate the regions which PCR primer pairs would amplify. (B) PCR was carried out to define the genomic region absent in a putative deletion line (Δ22) generated by remobilisation of the P-element k09932. Genomic DNA was prepared from a male fly with Δ22 over the deficiency Df(2L)ED125 lacking the entire region surrounding the kebab gene, together with a wild-type control (w) and other putative deletions. PCR was carried out using each primer pair shown in A. The regions b and c are missing from Δ22, but a and d are intact. (TIF) [file pone.0024174.s005.tif]
